# Initial Dose Tarlatamab–Associated Tumour Lysis Syndrome in Small Cell Lung Cancer: A Case Report

**DOI:** 10.1002/rcr2.70667

**Published:** 2026-06-28

**Authors:** Katsuhiko Shibata, Hiromu Tanaka, Yusuke Arai, Yusuke Watanuki, Takashi Kokubo, Yo Watanabe, Shingo Nakayama, Takahiro Asami, Takashi Inoue

**Affiliations:** ^1^ Department of Internal Medicine Sano Kosei General Hospital Tochigi Japan; ^2^ Division of Pulmonary Medicine, Department of Medicine Keio University School of Medicine Tokyo Japan; ^3^ Division of Infectious Diseases and Respiratory Medicine, Department of Internal Medicine National Defense Medical College Saitama Japan; ^4^ Department of Infectious Diseases Keio University School of Medicine Tokyo Japan

**Keywords:** cytokine storm syndrome, immune checkpoint blockade, immune tolerance, small‐cell lung carcinoma, tumour lysis syndrome

## Abstract

Tumour lysis syndrome (TLS) in solid tumours is rare but may occur in small‐cell lung cancer (SCLC) with a high tumour burden. We report a case of fatal TLS following tarlatamab treatment for relapsed SCLC. A 61‐year‐old woman with limited‐stage SCLC developed recurrence with extensive liver metastases after chemoradiotherapy and durvalumab consolidation therapy. Owing to rapid disease progression, an initial 1‐mg step‐up dose of tarlatamab was administered as second‐line treatment. On Day 2, the patient developed mild cytokine release syndrome, which improved with dexamethasone. On Day 6, she suddenly developed respiratory failure and impaired consciousness. Laboratory findings, including elevated lactate dehydrogenase, potassium, phosphorus and uric acid levels with acute kidney injury, suggested TLS and she died later that day. This case demonstrates that tarlatamab can induce fatal TLS even after a single 1‐mg dose. Patients with SCLC with extensive liver metastases require careful monitoring and aggressive strategies for TLS prevention.

## Graphical abstract

We report a fatal case of acute TLS following a single 1‐mg dose of tarlatamab in a patient with relapsed SCLC with extensive liver metastases. This case highlights the importance of TLS risk assessment prior to tarlatamab treatment. Extensive liver metastases and elevated lactate dehydrogenase levels may be risk factors for TLS in patients with SCLC and clinicians should recognise that TLS can occur even after a single 1‐mg dose and consider prolonged monitoring and preventive strategies in high‐risk patients.

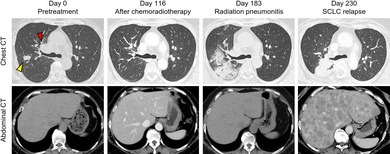

## 1 Introduction

Small‐cell lung cancer (SCLC) is an aggressive malignancy characterised by rapid progression, frequent relapse and poor prognosis. Tarlatamab, recently approved for relapsed SCLC treatment, is a bispecific T‐cell engager (BiTE) that binds to delta‐like ligand 3 on tumour cells and CD3 on cytotoxic T cells, inducing T‐cell‐mediated tumour cell lysis. Cytokine release syndrome (CRS) and immune effector cell‐associated neurotoxicity syndrome (ICANS) are major adverse events [1] that are managed using steroids and tocilizumab.

Tumour lysis syndrome (TLS) is a life‐threatening complication of cancer treatment caused by the rapid release of intracellular contents from lysed tumour cells, resulting in hyperuricemia, hyperphosphatemia and hyperkalemia, often followed by acute kidney injury and multiorgan failure. Risk factors include high tumour burden, elevated lactate dehydrogenase levels and preexisting renal dysfunction. However, TLS in solid tumours is rare [2] and risk factors for TLS remain underreported.

We report a fatal case of acute TLS following a single 1‐mg dose of tarlatamab in a patient with relapsed SCLC with extensive liver metastases, highlighting the importance of TLS risk assessment and early monitoring at treatment initiation.

## 2 Case Report

**FIGURE 1:**
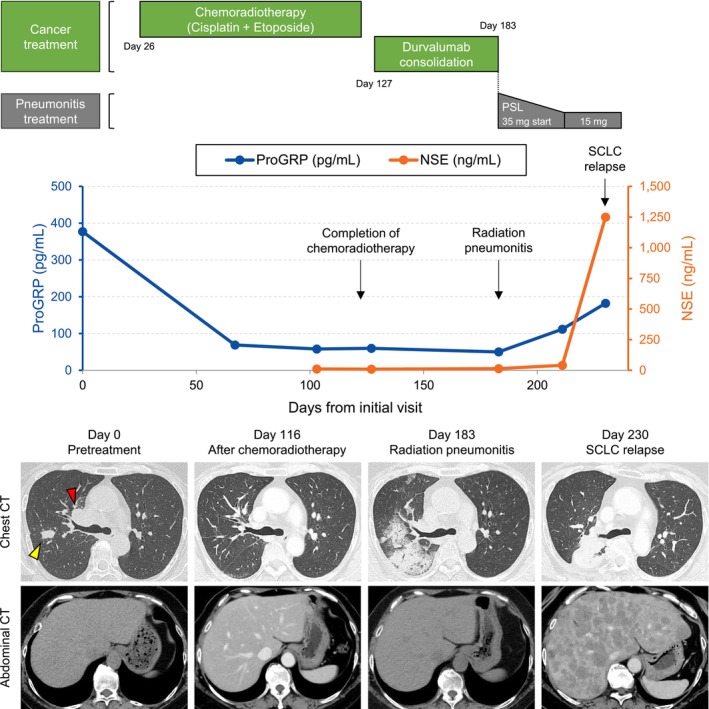
Timeline of the patient's clinical course before admission, showing serial changes in tumour marker (neuron‐specific enolase and pro‐gastrin releasing peptide) levels and computed tomography findings. Red arrows indicate the primary mediastinal/hilar lung cancer lesion and yellow arrows indicate pulmonary nodules suspected to represent intrapulmonary metastases. Abdominal CT images on Days 0 and 183 are without contrast, whereas those on Days 116 and 230 are contrast‐enhanced. Abbreviations: CT, computed tomography; NSE, neuron‐specific enolase; ProGRP, pro‐gastrin releasing peptide; PSL, prednisolone; SCLC, small cell lung cancer.

A 61‐year‐old woman was referred to our hospital for evaluation of abnormalities detected on a chest radiograph during a health checkup. She was a current smoker (20 cigarettes/day for 41 years) with a history of dyslipidemia. Chest computed tomography revealed bulky right‐sided hilar, mediastinal and supraclavicular lymphadenopathy and a right upper lobe pulmonary nodule considered to represent intrapulmonary metastasis. Bronchoscopic biopsy led to a diagnosis of limited‐stage SCLC (cT4N3M0, stage IIIC). She received concurrent chemoradiotherapy with cisplatin plus etoposide (4 cycles) and thoracic radiotherapy (accelerated hyperfractionation, 45 Gy/30 fractions), followed by durvalumab consolidation therapy. However, durvalumab was discontinued after two cycles because of radiation pneumonitis. The pneumonitis improved with prednisolone 35 mg/day, which was tapered to 15 mg/day (Figure 1). Subsequently, tumour marker elevation (neuron‐specific enolase, 1250 ng/mL; pro‐gastrin releasing peptide, 181.8 pg/mL) and extensive liver metastases were detected, suggesting recurrent SCLC.

**FIGURE 2:**
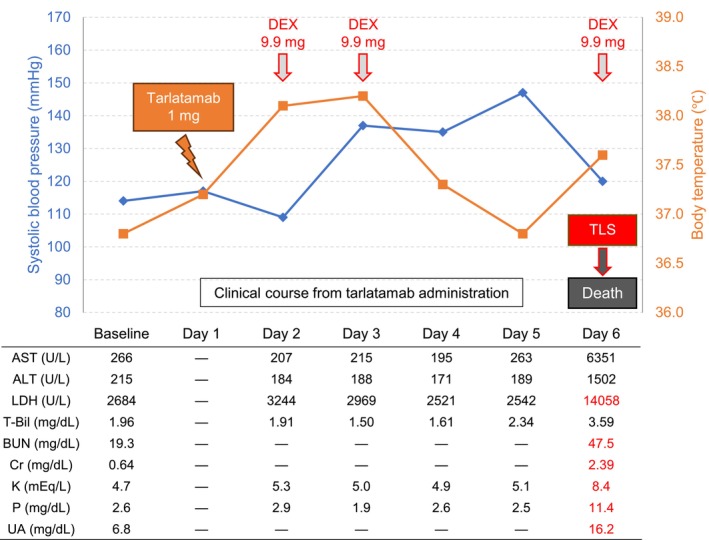
Timeline of the patient's clinical course after admission and following tarlatamab administration, showing serial changes in body temperature. systolic blood pressure and laboratory data indicating hepatic dysfunction and tumour lysis syndrome. Tarlatamab (1 mg) was administered on Day 1. Dexamethasone was administered for cytokine release syndrome on Days 2, 3 and 6. Abbreviations: ALT, alanine aminotransferase; AST, aspartate aminotransferase; BUN, blood urea nitrogen; Cr, creatinine; DEX, dexamethasone; K, potassium; LDH, lactate dehydrogenase; P, phosphorus; T‐Bil, total bilirubin; TLS, tumour lysis syndrome; UA, uric acid.

The patient was admitted for further treatment. On admission, liver function tests revealed hepatic failure (Figure 2). Owing to the rapid progression of liver metastases, tarlatamab was initiated as second‐line treatment (1 mg on Day 1). She developed fever and fatigue on Day 2, which was diagnosed as CRS and treated with dexamethasone, resulting in symptom improvement. Her condition remained stable on Days 4 and 5. However, on Day 6, she developed acute respiratory failure and impaired consciousness. Laboratory findings showed marked deterioration compared with the previous day's findings, including elevated lactate dehydrogenase (14,058 U/L), potassium (8.4 mEq/L), phosphorus (11.4 mg/dL) and uric acid levels (16.2 mg/dL), accompanied by acute kidney injury. Based on the Cairo–Bishop criteria [3], hyperkalemia, hyperphosphatemia and hyperuricemia within 7 days after tarlatamab initiation fulfilled the criteria for laboratory TLS, and the accompanying acute kidney injury established the diagnosis of clinical TLS. The respiratory failure rapidly worsened, despite dexamethasone treatment for suspected CRS, and she died later that day.

## 3 Discussion

Tarlatamab has changed the management of relapsed SCLC. However, its adverse‐event profile remains incompletely characterised. This case illustrates that tarlatamab can induce acute, fatal TLS even after a single dose and that extensive liver metastases may be a risk factor for TLS. Clinicians should therefore be aware of the risk of TLS when administering BiTE therapies to patients with bulky SCLC, particularly those with extensive liver metastases.

In this case, the occurrence of fatal TLS after a single 1‐mg dose of tarlatamab was likely due to rapid tumour cell destruction. In a clinical trial, CRS, fatigue and ICANS were the most common adverse events, whereas TLS was reported as an adverse event in only one patient [1]. Pharmacovigilance analyses indicate that immune checkpoint inhibitor (ICI)‐associated TLS is often severe and common in lung cancer, with a reported median time to onset of 9 days [4]. Although ICIs enhance antitumor immunity by releasing inhibitory checkpoints and may require days to weeks to generate clinically evident immune activation, tarlatamab directly bridges CD3‐positive T cells and DLL3‐expressing tumour cells. Given that DLL3 is commonly expressed in SCLC [5], this BiTE‐mediated engagement may efficiently redirect T cells towards SCLC cells, enabling T‐cell–mediated cytotoxicity with less dependence on endogenous antigen presentation or pre‐existing antitumor T‐cell responses. This mechanism can induce rapid T‐cell activation, cytokine release and cytotoxic lysis of SCLC cells, potentially resulting in fulminant TLS even after the initial 1‐mg step‐up dose. To our knowledge, only two cases of tarlatamab‐associated TLS have been reported previously and both had fatal outcomes [6]. One patient developed TLS on Day 3 after receiving a single 1‐mg dose, similar to our patient, suggesting that the initial step‐up dose may have induced sufficient antitumor activity to trigger TLS. In our case, CRS development shortly after tarlatamab administration further supports the notion that T‐cell activation had already occurred early after the initial dose. Therefore, even a single dose of tarlatamab can trigger fatal TLS.

TLS in solid tumours is generally rare. Most reported cases occur in metastatic disease, particularly lung cancer. Patients with SCLC are susceptible to TLS because of a high tumour burden, extensive metastases and marked treatment sensitivity. Extensive liver metastases are associated with a high tumour burden and elevated lactate dehydrogenase levels and hepatic dysfunction may accelerate metabolic deterioration once TLS develops. Given that previously reported cases of both tarlatamab‐ and nivolumab‐associated TLS in SCLC occurred in patients with extensive liver metastases [6, 7], this clinical feature should be recognised as a potential high‐risk factor for TLS. Moreover, this case also highlights the challenges associated with TLS monitoring. Immune‐mediated TLS may occur later than chemotherapy‐related TLS and can develop beyond the standard 48‐h monitoring period for CRS [4, 6]. Interestingly, our patient developed fulminant TLS on Day 6, despite no laboratory evidence being present on Day 5. This finding suggests that the abrupt onset of TLS may delay its recognition, despite laboratory parameters being carefully monitored. Therefore, in high‐risk patients, prolonged TLS monitoring should be performed in addition to CRS monitoring, and proactive TLS prevention, including aggressive hydration and uric acid‐lowering therapy, may need to be considered given the challenges of detecting the abrupt onset of TLS by monitoring alone.

This case highlights the importance of TLS risk assessment prior to tarlatamab treatment. Extensive liver metastases and elevated lactate dehydrogenase levels may be risk factors for TLS in patients with SCLC. Clinicians should recognise that TLS can occur even after a single 1‐mg dose and consider prolonged monitoring and preventive strategies in high‐risk patients.

## Author Contributions

Katsuhiko Shibata and Hiromu Tanaka contributed to the conception of this case report and drafted the original manuscript. Katsuhiko Shibata, Hiromu Tanaka, Yusuke Arai, Yusuke Watanuki, Takashi Kokubo, Yo Watanabe and Shingo Nakayama contributed to data interpretation and clinical assessment. Takahiro Asami and Takashi Inoue provided expert advice for clinical management and reviewed the manuscript. All authors read and approved the final version of the manuscript.

## Consent

Written informed consent for publication was obtained from the patient using the official Respirology Case Reports patient consent form.

## Conflicts of Interest

The authors declare no conflicts of interest.

## Data Availability

Data sharing not applicable to this article as no datasets were generated or analysed during the current study.
